# 1386. Prescribing Patterns of Skin and Soft Tissue Infections in Patients Discharged from the Emergency Department

**DOI:** 10.1093/ofid/ofac492.1215

**Published:** 2022-12-15

**Authors:** Jacqueline Feist, Nicholas Panecaldo, Pramodini Kale-Pradhan, George Delgado, Christopher Giuliano, Leonard B Johnson

**Affiliations:** Wayne State University Eugene Applebaum College of Pharmacy and Health Sciences, Detroit, Michigan; Wayne State University Eugene Applebaum College of Pharmacy and Health Science, Detroit, Michigan; Wayne State University Eugene Applebaum College of Pharmacy and Health Sciences, Detroit, Michigan; Ascension St. John Hospital, Detroit, Michigan; Wayne State University Eugene Applebaum College of Pharmacy and Health Sciences, Detroit, Michigan; Ascension St. John Hospital, Detroit, Michigan

## Abstract

**Background:**

Treatment of skin and soft tissue infections (SSTIs) are a growing reason for Emergency Department (ED) visits. Inadequate treatment of SSTIs has been reported in up to 25% of patients treated in ED. The purpose of this project was to describe prescribing patterns for SSTIs from the ED.

**Methods:**

Single center retrospective cohort study of SSTI patients discharged from ED between July 1- December 31, 2020. The primary outcome was to describe the proportion of patients receiving guideline directed therapy (GDT) for SSTIs. Secondary outcomes include describing the proportion of patients readmitted within 30 days, the reason for readmission, and to compare 30-day readmission rates in patients who received GDT versus non-GDT.

**Results:**

Of 876 patients screened, 576 were excluded primarily for being admitted to the hospital. Of the 300 included patients, 117 (39%) received GDT and 183 (61%) received non-GDT. Fifty-six of 300 patients were readmitted within 30 days, of which 21 (17.9%) had received GDT and 35(19.1%) had received non-GDT (p=0.8). Readmissions related to persistence or progression of SSTI were not significantly different between GDT and non-GDT, 9 patients and 11 patients respectively (p=0.57). The remaining 36 patients (64.3%) were readmitted for reasons unrelated to SSTI.

**Conclusion:**

The majority of prescribed antibiotic therapy for SSTI did not conform to GDT. However, 30-day readmission rates were not significantly different between the groups. Further research on GDT and ED treated SSTI is needed.

**Disclosures:**

**All Authors**: No reported disclosures.

